# A Population Description of Young Women with Breast Cancer in Newfoundland and Labrador

**DOI:** 10.3390/curroncol30110695

**Published:** 2023-10-31

**Authors:** Meghan Mahoney, Saranga Sriranganathan, Jeff Dowden, Melanie Seal

**Affiliations:** 1 Discipline of Oncology, St. John’s, NL A1B 3V6, Canada; memahoney@mun.ca; 2 Faculty of Medicine, Memorial University, St. John’s, NL A1B 3V6, Canada; ssriranganat@mun.ca; 3 Cancer Care Program, Newfoundland & Labrador Health Services (NLHS), St. John’s, NL A1B 3V6, Canada; jeff.dowden@easternhealth.ca

**Keywords:** breast, cancer, young, women cohort, subtype, survival, population, description

## Abstract

It has been well established in the literature that young women tend to carry more aggressive subtypes of breast cancer than their older-aged counterparts. The objective of this study was to describe the characteristics and outcomes of young women with breast cancer. In this retrospective analysis, data were collected for women under the age of 40 years who were diagnosed with breast cancer between 2008 and 2018 in the province of Newfoundland and Labrador. Specifically, data were collected on demographics, staging, pathological characteristics, treatment, and survival outcomes for young women with this disease. Results demonstrate that most of these women were diagnosed between the age of 35 and 39 years (91.2%). Most women presented with early-stage disease (stage I and II—66.4%), while 24% were stage III and 9.6% presented with stage IV metastatic disease. The prevalence of hormone-receptor-positive disease represented 41.9% of the cohort, with triple-negative and HER2+ measuring 27.7% and 30.4%, respectively. Five-year disease-free survival was 80.5% and overall survival was 82.7%. These findings provoke discussion regarding the intersecting roles of genetics, environment, and lifestyle in a region with some of the highest rates of malignancy in the country.

## 1. Introduction

Breast cancer is the most commonly diagnosed malignancy amongst women in Canada, with incidence rates that increase with age [1]. Young women with breast cancer, or those diagnosed before age 40, represent a small but significant subset of this population that present with unique disease characteristics and management challenges. While women under 40 represent only 4% of breast cancer diagnoses [2], female breast cancer is the most common malignancy amongst adolescents and young adults aged 15–39 [3]. Breast cancer in this population tends to present at a later stage than it does for older females with a more aggressive tumor biology carrying significant morbidity and mortality rates [4]. Young women with breast cancer have higher rates of both local and distant recurrences as well as lower survival rates when compared to older adults [5]. Even amongst those who present with early-stage disease, young women have been found to be almost 40% more likely to die from breast cancer compared to those over 40 years [6]. This has led to the establishment of specific guidelines for the care of adolescents and young adults with breast cancer in recent years and highlights the importance of high-quality data and evidence to inform best practices [7]. 

The province of Newfoundland and Labrador was projected to have the highest age-standardized incidence rates (ASIRs) in the country for females in 2021 (542.9 per 100,000). Rates of breast cancer in females specifically are expected to be higher in Newfoundland and Labrador than any other province in Canada (136.6 per 100,000 in Newfoundland and Labrador versus 126.8 per 100,000 within Canada) [1].

Breast cancer is a heterogeneous disease consisting of specific biological and molecular subtypes. The expressions of estrogen receptor (ER), progesterone receptor (PR), and human epidermal growth factor receptor 2 (HER2) are molecular markers that classify breast cancer subtype, determine the prognosis, and carry significant implications for treatment across all age groups. Tumors that do not express hormone receptors but express HER2, or do not express any receptors at all (also referred to as ‘triple negative’), demonstrate more aggressive phenotypes and are known to lead to inferior outcomes and lower overall survival [8]. It has been well established in the literature that a greater proportion of young women carry these higher-risk subtypes [9]. In addition, tumors that present in younger women are more likely to be a higher grade with more proliferative capacity and increased vascular invasion [10]. 

It should be noted that even when young patients with breast cancer are matched according to biomarker subtypes and compared to older women, they continue to demonstrate worse survival outcomes [11]. Reasons for this discrepancy are thought to be attributable to further biological and non-biological factors. Young women who are diagnosed with breast cancer are more likely to carry familial genetic mutations, which predispose them to developing the disease at an earlier age [12]. One study estimated that half of women under the age of 30 diagnosed with breast cancer have germline mutations in BRCA1, BRCA2, or TP53 [13]. Women who are genetic carriers of these mutations are also at an increased lifetime risk of developing other malignancies, and their mutational status carries implications that impact both surgical and medical treatment decisions [14]. 

Beyond biological and lifestyle risk factors, young women present with complex challenges in the face of breast cancer diagnoses that are unique to this cohort. Screening too plays a role. While the Canadian Task Force on Preventative Health Care currently recommends mammography for women starting at the age of 50, there is no screening recommendation for average-risk young women as benefits have not been found to outweigh risks before age 49 [15], and breast masses in young patients are often identified with self-examination [16]. While screening protocols exist for patients who are considered high risk (i.e., known carrier of a BRCA1/2, strong family history, or previous thoracic radiation), average-risk young females are not routinely screened for breast cancer [15], potentially contributing to their later-stage diagnoses. Adolescents and young adults with breast cancer report notably higher impacts on their quality of life compared to older women [17] as issues including fertility, body image, and psychosocial factors make treatment and survivorship particularly cumbersome to navigate. It is for this reason that current consensus guidelines emphasize the importance of multimodality care with collaboration between their physicians and allied healthcare team. 

The objective of this study is to describe the demographic, pathological, and survival outcomes of young women (<40 years) with breast cancer in Newfoundland and Labrador. The aim is to contribute to the literature on this unique and often underrepresented population in the province projected to carry the highest rates of breast cancer in Canada. The analysis of these data sparks discussion pertaining to the intersection of genetic and environmental risk factors calling for careful consideration of the trends demonstrated in this young patient cohort.

## 2. Materials and Methods

A retrospective chart review was performed to obtain population-based data pertaining to young women with breast cancer in the province of Newfoundland and Labrador. Women aged <40 years old at the time of diagnosis between 2008 and 2018 were included. They were required to have had biopsy-proven, invasive breast cancer (i.e., patients with ductal carcinoma in situ (DCIS) were excluded), which was managed within the province. Following ethics approval, patients were identified within the Newfoundland and Labrador Cancer Care Registry (NLCCR) and data were abstracted from electronic medical records. The initial data extraction included 158 patients with 148 patients meeting inclusion criteria after chart review was performed. The ten excluded patients were those who were treated primarily out of province or who did not have an invasive breast cancer diagnosis. Study variables related to patient information that were analyzed during this chart audit included demographic (age at diagnosis, BMI as categorized by the World Health Organization), pathological (tumor stage, grade, and subtype), and treatment-related variables (i.e., neoadjuvant therapy, ovarian function suppression, and up-front surgery). Survival data were collected, including 5-year disease-free and overall survival. Disease-free survival was defined as the absence of local or distant recurrence of breast cancer, or the presence of a new primary 5 years out from their date of diagnosis, while overall survival captured mortality at this timepoint.

The electronic database was password-protected and deidentified. Data collection was completed with retrospective chart review by M.M. and S.S. Statistical analyses were performed using the statistical analysis software (Statistical Package for the Social Sciences, IBM SPSS Advanced Statistics 28.0.1.0 (5725-A54)) to identify notable trends and descriptive statistics for this patient population. Specifically, frequency analyses and crosstabs were utilized to yield proportions and percentages for the data variables.

## 3. Results

Data were collected on 148 patients who met eligibility criteria and frequency statistics pertaining to each of the variables of interest can be found in Table 1. The majority of patients were within the 35–39 age group (135 patients, 91.2%). Most women in this cohort demonstrated early-stage disease at the diagnosis (stage I or II disease), while 9.6% (14 patients) presented with stage IV, de novo metastatic disease. Biomarkers were noted for each patient, with 62 patients (41.9%) being hormone-receptor-positive (positive for estrogen, progesterone, or both and HER2-negative) and 45 (30.4%) patients had HER2-receptor-positive disease (which was inclusive of those testing positive for hormone receptors in addition to HER2). Triple-negative disease was reported in 41 patients, 27.7% of cases. 

The body mass index (BMI) was recorded at the time of the diagnosis. Most patients in this cohort (60.5%) fell into categories of overweight (25.2% of patents with BMI between 25.0 and 29.9) or obese (35.3% of patients with BMI greater than 30).

With regards to initial surgical management, 56% of women underwent mastectomy and 29.8% had breast-conserving surgery. Thirty-two patients (21.6%) were treated with systemic therapy prior to their initial surgery (neoadjuvant setting) and ovarian function suppression was delivered to 58 patients (41.7%).

**Figure 1:**
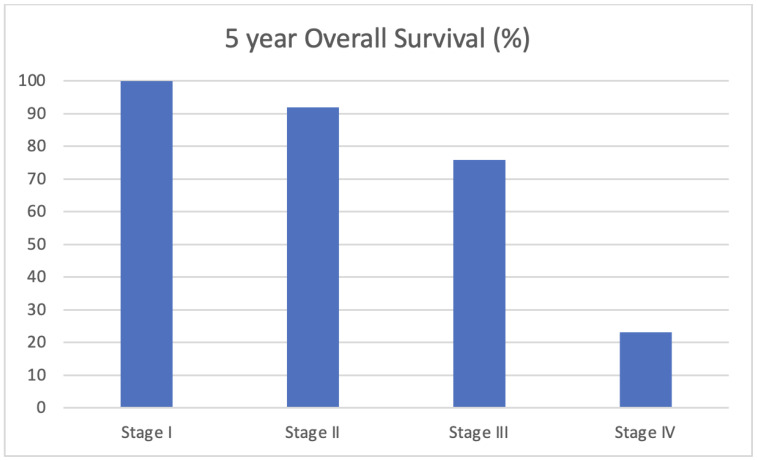
Five-year overall survival by stage at diagnosis in young women (<40 years) with breast cancer in Newfoundland and Labrador.

**Figure 2:**
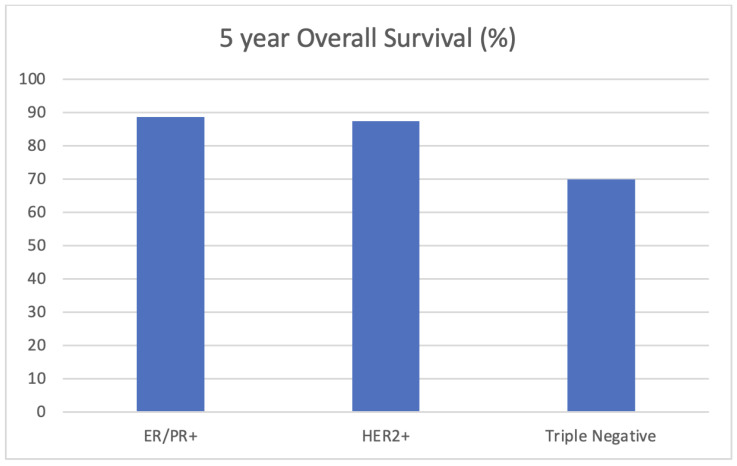
Five-year overall survival by biomarker subtype in young women (<40 years) with breast cancer in Newfoundland and Labrador.

**Figure 3:**
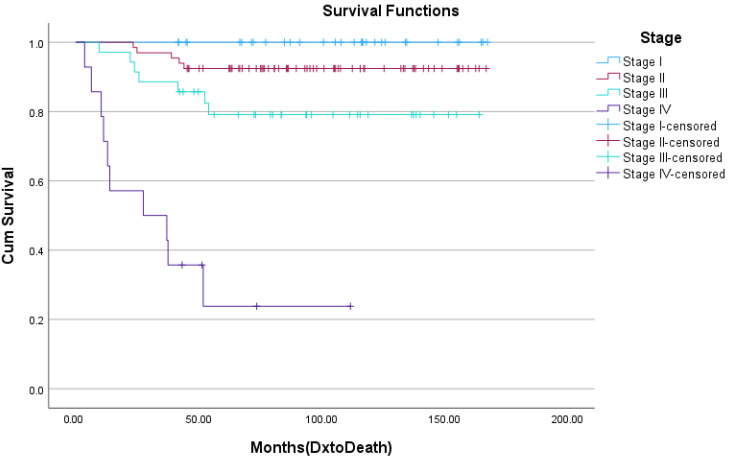
Kaplan Meier curve of 5-year overall survival by cancer stage at diagnosis.

**Figure 4:**
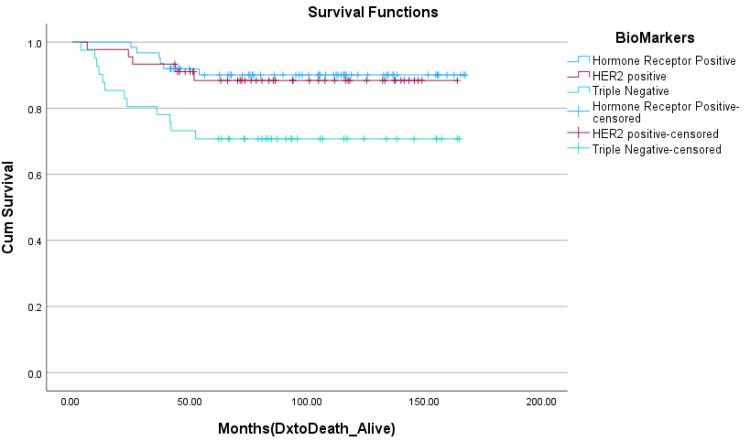
Kaplan Meier curve of 5-year overall survival by biomarkers at diagnosis.

Five-year disease-free survival and overall survival data were collected for this cohort with the exception of patients diagnosed in 2018 who did not meet this 5-year mark at the time of the analysis. At 5 years, 80.5% of women were disease-free, with no locoregional or systemic recurrences. Five-year overall survival was 82.7%. As demonstrated in Figure 1, the 5-year survival rates were 88.7% for women with hormone-receptor-positive disease, 87.5% for women with HER2-positive disease, and 70.0% for those with triple-negative breast cancer. Figure 2 depicts overall survival by stage at the diagnosis. Those with early-stage disease demonstrated better overall survival at 5 years, while overall survival was only 23.1% for those who had presented with stage IV, metastatic breast cancer. Kaplan Meier survival curves for stage and biomarker status are depicted in Figure 3 and Figure 4, respectively. 

## 4. Discussion

In this study of young women with breast cancer in the province of Newfoundland and Labrador, patient demographics, tumor biology, and survival outcomes were evaluated. Several key themes emerged from these data prompting comparison to similar patient cohorts. Points of discussion include prevalence of biomarker subtypes, BMI, and survival data. 

As demonstrated, the prevalence of HER2+ disease and triple-negative disease, the more aggressive biological subtypes of breast cancer, was 30.4% and 27.7%, respectively, in the Newfoundland and Labrador population. A large, North American cohort (*n* = 46,265) that was evaluated by Murphy et al. of young women under the age of 40 with breast cancer diagnosed between 2010 and 2015 reported rates of HER2+ disease of 26.0% and triple-negative disease of 21.2% [18]. A population of young women with breast cancer in France reported a rate of triple-negative disease of 19.1% amongst those aged 35–40, with a HER2+ rate of 16.4% [10]. The rates of these aggressive tumor subtypes are notably higher in the Newfoundland and Labrador population of young women with breast cancer. Reasons for this discrepancy may be considered within the context of both genetic and environmental factors. It has been well established that Newfoundland and Labrador genetics stem from a limited founder population, as those of English and Irish decent in the mid-1700s comprise the genetic backbone for the majority of the inhabitants. Scientific medical data from the province have therefore been an attractive resource in the evaluation of complex diseases harboring genetic mutations. A founder effect in Newfoundland and Labrador has been identified in the prevalence of several malignancy-associated Mendelian disorders such as MEN1 (multiple endocrine neoplasia type 1) and HNPCC (hereditary non-polyposis colorectal cancer, also known as Lynch Syndrome) [19]. It has therefore been suggested that the Newfoundland and Labrador young female population may carry a higher prevalence of genetic mutations, carrying an increased risk for breast cancer such as BRCA1/2, predisposing them to these more aggressive tumor biologies, such as triple-negative disease.

With regards to modifiable risk factors, our cohort was noted to carry a significant burden of obesity at the time of the diagnosis, with 60.5% of patients falling into categories of overweight (25.2%) or obese (35.3%). Newfoundland and Labrador carries the highest provincial obesity rate in the country, with 42% of adults having increased adiposity as of 2021 [20]. This may be hypothesized to contribute to the higher rates of aggressive tumor biology seen within this study population. It should be noted however that there is debate surrounding the association between body weight/body mass index (BMI) and risk of a breast cancer diagnosis in young females. Obesity is known to increase breast cancer risk for older women; however, some studies have associated low to moderate BMIs with an increased risk of breast cancer in younger populations. More recent studies evaluating waist circumference however have found central obesity to increase risk in both pre- and postmenopausal populations [21]. Increased physical activity, low red meat consumption, and plant-based diets have been associated with a lower risk of breast cancer in the AYA population; however, this is based on limited data from small studies and a further analysis with large, multicenter prospective trials is required [22]. There is variability pertaining to the contributory role of other modifiable risk factors for breast cancers as well. For example, while the association between alcohol intake and an increased risk of developing breast cancer in older populations is robust, the role of alcohol as a modifiable risk factor for young patients is unclear [23], reiterating that the role of modifiable risk factors warrants further investigation in young females with breast cancer.

The analysis of survival outcomes demonstrated that 80.5% of patients were disease-free without systemic or locoregional recurrences at 5 years, while the overall survival for this patient population was 82.7%. These represent lower overall survival compared to similar patient cohorts. The American National Cancer Institute (NCI) reports a 5-year overall survival rate of 86.4% amongst female breast cancer patients aged 15–39 between 2013 and 2019 [24]. The aforementioned French population study on young women diagnosed with breast cancer between 1980 and 2014 found the overall survival for women between the ages of 35 and 40 to be 92.3% at 5 years [10]. That study noted a 5-year disease-free survival rate of 87.6%, compared to the 80.5% found within this study. When broken down by biomarker status, the 5-year overall survival for the Newfoundland and Labrador cohort for hormone-receptor-positive, HER2+, and triple-negative breast cancers was 88.7%, 87.5%, and 70.0%, respectively, reflecting worse outcomes for more aggressive disease pathologies as anticipated. The slightly higher rates of those subtypes (HER2+ and triple-negative breast cancer) in the province may account for the lower 5-year overall survival rate when compared to other North American populations. 

A notable limitation in this study is the lack of data collection on specific chemotherapy regimens received by each patient. This was in part inaccessible as systemic therapy regimens were available from various EMR systems for some patients and not others. It is not believed however that a lack of access to care was a factor in outcomes and it is presumed that patients were treated according to the standard of care at the time. Another limitation of this study includes the lack of access to universal genetic mutation status amongst this cohort. While some patients had documented mutations, it was not clear whether the entire cohort had undergone genetic assessment. As per the current provincial guidelines (see supplementary materials), any young female diagnosed with breast cancer prior to age 40 warrants a referral to genetics and it is hoped that these data will be available in this regard in the near future. This is of notable importance as mutational status may warrant changes in systemic therapy due to recent advancements in the realm of treatment of BRCA mutated breast cancer with PARP (poly-ADP ribose polymerase protein) inhibitors. Other limitations warrant considerations in guideline protocols and standards of care, which evolved within the global landscape of breast cancer treatment during the study time period. For example, the indications for neoadjuvant systemic therapy or ovarian function suppression, which were incorporated into guidelines in the last several years, were not the standard practice nationally or provincially during the earlier years of this study, which may account for variations in the data. It is imperative that future research in this area explores comprehensive aspects of these patients’ care including geographic location (urban versus rural), fertility, mental health and wellness, as well as psychosocial supports. 

This population description of young women with breast cancer in the province of Newfoundland and Labrador has produced insights into the demographics and outcomes of an often underrepresented and vulnerable population in the province projected to have the highest incidence rates of female breast cancer in Canada. The high proportions of aggressive subtypes of breast cancer and the reduced overall survival associated with those diagnoses that have been demonstrated in this cohort highlight the importance of multidisciplinary care and attention to guidelines, particularly with respect to genetic testing. Detection of genetic mutations influences treatment decisions pertaining to systemic therapy, impacting surgical decision making and family planning. As a result, early genetic testing and referral is essential for young, high-risk women. Fertility considerations in this young cohort are another area that could avail from further assessment given the difficulties that these patients often face when trying to conceive. Most notably, however, this study calls for an extensive investigation into the epigenetic implications within the province of Newfoundland and Labrador that conceivably contribute to higher rates of more aggressive malignancies. This unique intersection of environmental and genetic risk factors may shed light upon pertinent pathways of oncogenes that provoke phenotypic expression of malignancies and that may provide investigators with the questions that must be asked in order to identify and best treat oncology patients.

## Figures and Tables

**Table 1:** Patient demographics, disease characteristics, management, and survival outcomes in women <40 years diagnosed with breast cancer between 2008 and 2018, by 5-year overall survival.

Variable	Percent Deceased at 5 Years (*n* = 23)	Percent Alive at 5 Years (*n* = 110)	*p*-Value (Chi-Square/Fisher’s Exact *)
Age			
25–30	0.0% (0)	1.8% (2)	0.751
30–35	8.7% (2)	6.4% (7)	
35–50	21% (21)	91.8% (101)	
BMI at diagnosis			
<18.5 (Underweight)	0.0% (0)	0.9% (1)	0.967
18.5–<25 (Normal)	26.1% (6)	27.3% (30)	
25–<30 (Overweight)	21.7% (5)	21.8% (24)	
30 or greater (Obese) Unknown	26.1% (6) 26.1% (6)	30.9% (34) 19.1% (21)	
Stage at diagnosis			
Stage I	0.0% (0)	24.5% (27)	≤0.05
Stage II	21.7% (5)	51.8% (57)	
Stage III	30.4% (7)	20.0% (22)	
Stage IV	43.5 (10)	2.7% (3)	
Unknown	4.3% (1)	0.9% (1)	
Tumour Grade			
I	0.0% (0)	7.3% (8)	0.218
II	21.7% (5)	34.5% (38)	
III	65.2% (15)	54.5% (60)	
Unknown	13.0% (3)	3.6% (4)	
Tumour subtype			
Triple-negative	52.2% (12)	25.5% (28)	0.039
HER2+	21.7 (5)	31.8 (35)	
HR+/HER2-	26.1% (6)	42.7% (47)	
Initial surgical management			
Mastectomy	77.8% (14)	51.9% (56)	0.121
Bilateral Mastectomy	5.6% (1)	14.8% (16)	
Lupectomy	16.7% (3)	33.3% (36)	
Neoadjuvant systemic therapy			
Yes	39.1 (9)	16.4% (18)	0.021
No	60.9 (14)	83.6% (92)	
Ovarian function suppression			
Yes	23.5% (4)	41.7% (45)	0.189
No	76.5% (13)	58.3% (63)	

* Fisher’s exact test performed when expected cell counts were less than 5.

## Data Availability

Data available on request due to restrictions. The data presented in this study are available on request from the corresponding author. The data are not publicly available due to privacy and ethical considerations, aside from data presented within the paper.

## Supplementary Materials

Newfoundland and Labrador current breast cancer guidelines: https://cancercare.easternhealth.ca/health-care-professionals/guidelines/breast-cancer/?perPage=25, accessed on 30 October 2023.
